# Signaling Activation and Modulation in Extrafollicular B Cell Responses

**DOI:** 10.1111/imr.70004

**Published:** 2025-02-07

**Authors:** Julian Staniek, Marta Rizzi

**Affiliations:** ^1^ Department of Rheumatology and Clinical Immunology, Faculty of Medicine, University Medical Center Freiburg University of Freiburg Freiburg Germany; ^2^ Faculty of Medicine, Center for Chronic Immunodeficiency, University Medical Center Freiburg University of Freiburg Freiburg Germany; ^3^ Division of Clinical and Experimental Immunology, Institute of Immunology, Center for Pathophysiology, Infectiology and Immunology Medical University of Vienna Vienna Austria; ^4^ CIBSS—Centre for Integrative Biological Signalling Studies University of Freiburg Freiburg Germany

**Keywords:** CD21low, extrafollicular response, FAS, mTOR, PI3K, signaling, T‐bet B cells

## Abstract

The differentiation of naive follicular B cells into either the germinal center (GC) or extrafollicular (EF) pathway plays a critical role in shaping the type, affinity, and longevity of effector B cells. This choice also governs the selection and survival of autoreactive B cells, influencing their potential to enter the memory compartment. During the first 2–3 days following antigen encounter, initially activated B cells integrate activating signals from T cells, Toll‐like receptors (TLRs), and cytokines, alongside inhibitory signals mediated by inhibitory receptors. This integration modulates the intensity of signaling, particularly of the PI3K/AKT/mTOR pathway, which plays a central role in guiding developmental decisions. These early signaling events determine whether B cells undergo GC maturation or differentiate rapidly into antibody‐secreting cells (ASCs) via the EF pathway. Dysregulation of these signaling pathways—whether through excessive activation or defective regulatory mechanisms—can disrupt the balance between GC and EF fates, predisposing individuals to autoimmunity. Accordingly, aberrant PI3K/AKT/mTOR signaling has been implicated in the defective selection of autoreactive B cells, increasing the risk of autoimmune disease. This review focuses on the signaling events in newly activated B cells, with an emphasis on the induction and regulation of the PI3K/AKT/mTOR pathway. It also highlights gaps in our understanding of how alternative B cell fates are regulated. Both the physiological context and the implications of inborn errors of immunity (IEIs) and complex autoimmune conditions will be discussed in this regard.

The primary repertoire and the naive B cell compartment ensure the ability to respond to virtually any pathogen or antigen, leading to the generation of memory B cells and short‐ or long‐lived plasma cells. Upon initial activation, B cells can rapidly develop via the extrafollicular (EF) pathway into antibody‐secreting cells (ASCs), which typically exhibit low affinity for the antigen, carry few somatic mutations, have a short lifespan and provide rapid protection against the pathogen. Later in time, or as an alternative pathway, B cells can migrate to the germinal center (GC), where they undergo somatic hypermutation (SHM) and affinity maturation. In this process, B cells with higher affinity for the antigen are selected to differentiate into long‐lived memory B cells and plasma cells, ensuring the establishment of specific and durable humoral memory. The developmental decision of a naive B cell to differentiate into specific effector fates is governed by the integration of signals from the B cell receptor (BCR), co‐stimulatory molecules, Toll‐like receptors (TLRs), cytokines and inhibitory signals. In addition to the nature of these stimuli, their precise temporal and spatial coordination is essential, ultimately guiding the fate of individual cells toward either the GC or EF response [1] (Figure 1). We recently discovered that the TNF receptor superfamily member FAS (CD95, Apo1) plays a role in the developmental decision between the EF and the GC response by regulating PI3K/AKT/mTOR activation in CD40‐activated human B cells [2]. Our discovery highlights the importance of regulatory receptors and the modulation of signaling in driving cell fate decisions.

**FIGURE 1:**
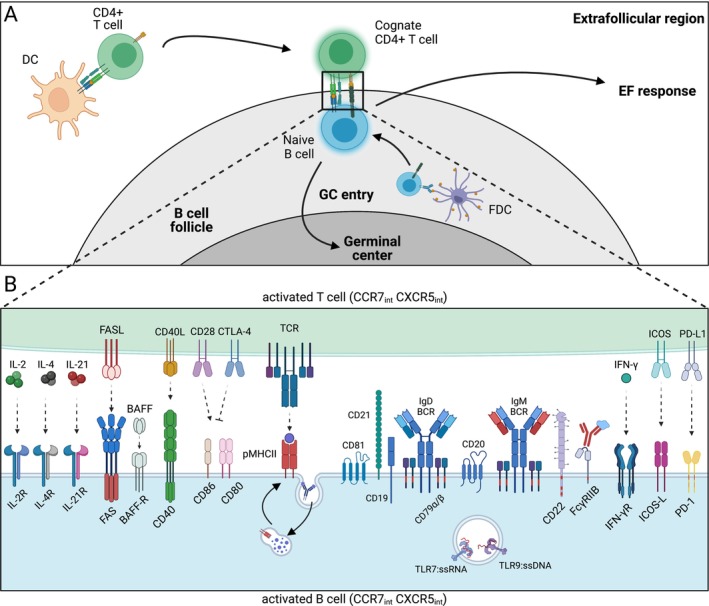
Signaling in B cells is mediated by activating receptors, which are integrated and regulated at multiple levels to guide specific cellular functions. (A) Upon activation by dendritic cells (DCs) in the interfollicular region, T cells migrate to the T‐B border, where they encounter cognate B cells that have engaged with the antigen exposed by follicular dendritic cells (FDCs). BCR activation and T‐B interaction determine whether activated B cells will enter the germinal center (GC) or differentiate at extrafollicular (EF) sites in the EF response. (B) During the initial activation, several receptors instruct B cell fate decisions. Naïve B cells bind to cognate antigen with IgM or IgD‐BCRs that are embedded in the BCR complex, including the signaling subunits CD79α and CD79β (encoding cytoplasmic ITAMs), CD19, CD21, CD20, and CD81. T‐B interaction during initial activation is characterized by several ligand‐receptor pairs including T cell receptor (TCR) and peptide–MHC Class II, CD40L and CD40, CD28/CTLA‐4 and CD80/CD86, ICOS and ICOS‐L, PD‐1 and PD‐L1, FASL and FAS, as well as cytokines and their respective receptors. TLR and BAFFR activation may occur independent of T cell help. Engagement of negative regulators like CD22, FcγRIIB, and FAS is crucial to fine‐tune B cell fate decisions.

In the current view, long‐lived memory B cells and plasma cells are primarily generated through the GC reaction [3]. Indeed, persistence of GCs correlates with the increased frequency of antigen‐specific long‐lived bone marrow plasma cells [4, 5, 6, 7]. The EF response is generally composed of B cells characterized by the expression of CD11c and the transcription factor T‐bet and low or absent expression of CD21. Based on their phenotype, these cells are described in the literature as atypical memory cells, CD21 low B cells, age‐associated B cells (ABCs), or Tbet+ B cells. While these populations may exhibit substantial overlap, they are not entirely identical, complicating the interpretation of clinical observations where their expansion is noted. In fact, expansion of CD21 low B cells may occur in different immunological settings, such as during antiviral responses, chronic infections, inborn errors of immunity (IEIs), immunodeficiency, and autoimmunity [8, 9]. Whenever possible, for the purpose of this review we use the nomenclature introduced by Sanz et al. to identify B cells associated with EF responses in human autoimmunity and infection [10, 11]. This nomenclature is based on CD11c, CD21, CD38, CD27, and surface immunoglobulin (Ig) expression and divides the populations in double‐negative (DN)1, 2, 3, activated naïve, resting naïve B cells and ASCs, and allows the identification of distinct effector subsets of the EF response (activated naïve, DN2, DN3, and a subpopulation of ASCs) with little to no redundancy and distinguishes them from B cells associated with the GC pathway. The EF response results in the generation of short‐lived, low‐mutated ASCs that can differentiate rapidly upon antigen encounter from their precursors (activated naïve and DN2 cells) and can be associated with the development of autoantibodies [12]. Notably, a subset of CD21 low B cells has also been described to express the canonical human memory B cell marker CD27 [13]. CD27+ CD21 low B cells, along with ABCs, are sometimes regarded as graduates of GCs [8], but as we are focusing on early activation events, this specific developmental pathway will not be discussed in detail here.

Upon entering secondary lymphoid organs (SLOs) and interacting repeatedly with cognate T cells over several days, naive B cells undergo dynamic shifts in receptor expression and migration patterns. Initially, B cells increase CCR7 expression and relocate to the interfollicular region in a manner dependent on CXCR5 and EBI2. As they commit to the GC, B cells decrease EBI2 expression, upregulate CXCR5, and proliferate extensively before migrating into the follicle. In contrast, EF‐destined B cells lose CXCR5 expression but retain EBI2 expression, guiding their migration and differentiation outside the follicle. Simultaneously, activated CD4+ T cells primed by dendritic cells differentiate into distinct subsets, including T follicular helper (TFH) cells, which are characterized by BCL6 and CXCR5 expression, allowing them to migrate to B cell follicles. Other T cells differentiate into effector subsets, some of which exit SLOs, while others localize to EF sites, where they provide support to B cells outside of GCs [1, 14]. Unlike T cells, which differentiate into a variety of effector subtypes, B cell differentiation is largely restricted to two main effector subsets: memory B cells or ASCs, each with distinct survival properties. The partial clonal overlap between these two compartments suggests two possibilities: first, that commitment to a developmental pathway can occur early, likely before substantial proliferation, and consequently, individual B cells commit to a single fate. Second, the presence of shared clones across different effector subsets implies that some cells have the potential to give rise to all subsets, supporting the idea of a later divergence in their developmental trajectories [15]. Consequently, the strength and specificity of antigen signaling may drive cells along the EF developmental pathway, whereas in other cases, the integration of signals from both activating and inhibitory receptors may influence GC commitment.

## 1 Class Switch Recombination and Somatic Hypermutation in EF Responses

Both class switch recombination (CSR) and SHM were traditionally thought to occur exclusively within GCs, where selection mechanisms involving TFH cells and antigen help safeguard the maintenance of self‐tolerance [16]. Indeed, pathogenic antibodies are almost always class‐switched and carry somatic mutations, features that are typically associated with a GC origin. However, recent studies suggest that both GCs and EF responses can be the origin for the break of B cell tolerance (reviewed here [17]). In fact, isotype switching may be more frequent at EF sites than previously assumed, occurring even before GC formation [18]. In addition, ASC differentiation in EF responses is in many models driven by TLR signaling and in some cases independent of T cell help [12, 19, 20]. Interestingly, the presence of SHM has been observed in autoreactive plasma cells developing at EF sites in spleens of FAS‐deficient MRL‐lpr mice [21, 22], where the mutation rate of EF responses was estimated to be comparable to that seen in peaking GC responses [23]. Similarly, somatic V region diversification and CSR have been detected in infections with dominant EF responses [12, 24, 25]. In agreement, formation of antinuclear antibodies has been observed in lupus‐prone mice in the absence of spontaneous GCs [20, 26, 27, 28] and autoantibody production is preserved in lupus‐prone mice deficient in GC formation [19, 29]. These observations collectively suggest that certain inflammatory environments suppress GC formation while simultaneously promoting EF differentiation, which, under such conditions, exhibit signs of SHM (reviewed here [30]). For example, severe cases of SARS‐CoV‐2 infection are associated with loss of GCs concomitant with a rapid expansion of the EF pathway [31, 32], and a *de novo* generation of self‐reactive antiviral IgG1 ASCs that show signs of low antigenic selection pressure with rare but detectable SHM [33]. In SLE patients, DN2 and DN3 B cells constitute a major population of antigen‐specific B cells after SARS‐CoV2 vaccination at priming and boosting [34]. Thus, in settings of autoimmunity or immunodeficiency, dysregulated control of GC versus EF commitment may result in a continuous irregular recruitment of naïve B cells with germline‐encoded autoreactivity into the EF pathway. However, additional studies are required to firmly establish whether SHM occurs at EF sites in autoimmune settings in the presence of GCs and, more generally, in ‘conventional’ immune responses with early formation of short‐lived plasma cells at EF sites and delayed but robust formation of GC‐derived memory B cells and long‐lived plasma cells. Finally, although TLR ligands can bolster EF responses and are required to induce autoimmunity in many situations [30], T cell help outside of GCs appears to be critical for the development of EF responses and autoimmunity [35]. In particular, EF TFH cells can drive B cell maturation outside of GCs [36, 37, 38, 39, 40], and additional peripheral helper T cell subsets have been identified, that may promote the formation of autoreactive B cells outside of GCs [41, 42, 43].

## 2 BCR Activation Strength Shapes B Cell Developmental Trajectory

**FIGURE 2:**
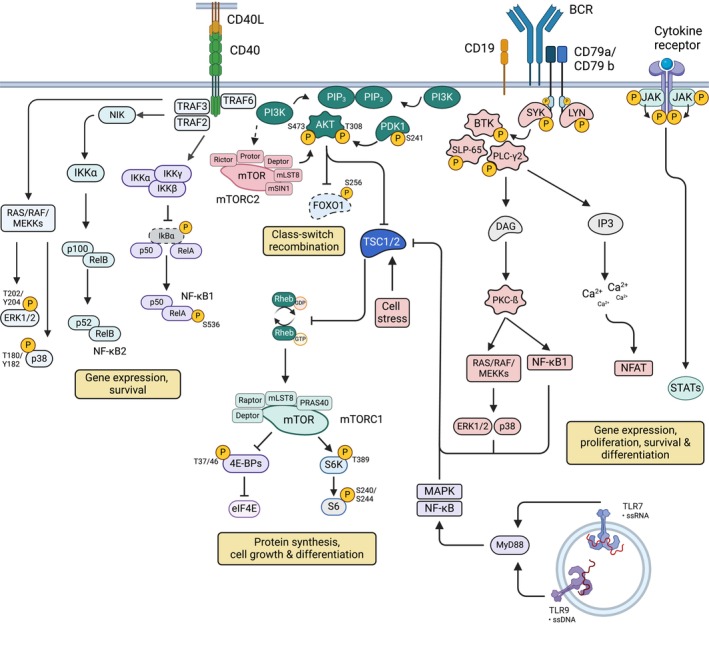
B cell signaling is integrated downstream of activating receptors to regulated B cell‐specific cellular functions and B cell fate decisions. Receptor engagement activates B cells, triggering multiple signaling pathways such as the canonical (NF‐κB1) and non‐canonical (NF‐κB2) NF‐κB pathways, along with MAP kinase pathways, which support gene expression and cell survival. B cell receptor (BCR) signaling also promotes gene expression, proliferation, and survival. Additionally, the activation of the mTOR signaling pathway, involving mTORC1 and mTORC2 complexes, facilitates protein synthesis, cell growth, and differentiation. Proteins associated with each signaling pathway are depicted in the figure. Examples of activating receptors are shown, with phosphorylation of specific intermediates highlighted in yellow, including the phosphorylated residues. Arrows indicate cross‐talk between different pathways, with the scheme primarily focusing on the activation of the PI3K/AKT/mTOR pathway.

The BCR serves a dual function as both a signaling molecule and an endocytic receptor. Consequently, the initial germline‐encoded antigen affinity, competition for antigen, and cognate T cell help are critical factors that determine the magnitude of B cell activation. Ligation of the IgM BCR in resting naive B cells results in reorganization of auto‐inhibitory BCR complexes. This nanoscale rearrangement reveals the immune receptor tyrosine‐based activation motifs (ITAMs) in the cytoplasmic domains of the BCR co‐receptors CD79a and CD79b and allows their phosphorylation by Src‐family kinases like LYN (Figure 2). CD79a/CD79b phosphorylation results in SH2‐domain‐mediated SYK recruitment and activation. BCR engagement also induces activation of phosphoinositide 3‐kinase (PI3K), which generates phosphatidylinositol‐3,4,5‐triphosphate (PI(3,4,5)P_3_) from phosphatidylinositol‐4,5‐bisphosphate (PI(4,5)P_2_). Membrane‐proximal PI(3,4,5)P_3_ production results in the recruitment of BTK and AKT to the plasma membrane via pleckstrin homology (PH) domains. This event results in BTK phosphorylation by SYK and the formation of a larger signaling scaffold consisting of BTK, SLP65 (also known as BLNK), and PLCγ2. In turn, BTK is fully activated and phosphorylates PLCγ2, promoting calcium influx and initiating several downstream BCR controlled signaling pathways, including NF‐κB and the MAPK pathway. (reviewed elsewhere [44]). AKT recruitment following BCR activation results in its phosphorylation, promoting the activation of the PI3K/AKT/mTOR pathway, described in more detail below. Naïve B cells co‐express both IgM and IgD‐BCRs. The function of IgD is not completely understood, since IgM expression is sufficient for the development, survival, and activation of B cells [45]. IgM and IgD‐BCRs have differential responsiveness to distinct types of antigens and IgD appears to modulate antigen‐specific B cell responses [46]. In fact, antigens with low valence activate only IgM‐BCRs, whereas polyvalent antigens trigger signaling through both IgM‐ and IgD‐BCR [47]. Further, autoreactive B cells specifically downregulate IgM‐BCRs but maintain expression of IgD‐BCRs, which are less sensitive to endogenous antigens. Such autoreactive IgM^low^ IgD^high^ B cells are prevented from rapid ASC differentiation at EF sites but can normally enter the GC [46]. Within the GC, through SHM and selection pressure, these cells may lose self‐reactivity, ultimately leading to clonal redemption [48, 49]. Upon T‐dependent vaccination with a multivalent antigen, IgD‐deficient mice display delayed GC formation and reduced antigen‐specific antibody titers compared to wild‐type mice. However, when immunized with a strong immunogen, both IgD‐deficient and wild‐type mice exhibit similar GC B cell formation. This suggests that the regulatory functions of IgD, and potentially other modulatory receptors, can be overridden under conditions of robust B cell activation [50]. The differential association of IgM and IgD‐BCRs with activating or inhibitory co‐receptors may fine‐tune BCR signaling, thereby influencing early B cell fate decisions [51]. In sum, depending on the nature of the antigen (hapten‐carrier versus natural protein epitope) and the density of the epitope on the antigen, BCR isotypes expressed by naive B cells may contribute to early B cell fate decisions. In this regard, IgD can be considered an inhibitory receptor, safeguarding reactivity to self‐antigens and modulating the intensity of BCR activation.

High‐affinity BCRs tend to favor EF differentiation [52, 53, 54, 55, 56, 57, 58], potentially due to stronger signaling that drives transcription factors like IRF4 [59] and, together with PI3K and mTOR activity, induces expression of Blimp1 to promote plasma cell differentiation [60, 61]. Hence, the substantial production of IgM‐secreting plasma cells in BCR transgenic mice immunized with distinct antigen affinities and avidities [56, 57, 62] may be a result of increased PI3K/AKT/mTOR activity and an accumulation of transcription factors like IRF4 and Blimp1 [61]. While the initial affinity of the BCR may not always directly dictate differentiation, it can enhance the proliferation of ASCs committed to EF differentiation [53].

## 3 The Role of T Cell Help, TLRs, and Cytokines

One of the critical signals provided by T cells in the initial B cell activation is mediated by CD40L. In B cells, CD40 recruits various TNF receptor‐associated factor (TRAF) proteins—TRAF1, TRAF2, TRAF3, TRAF5, and TRAF6—either directly or indirectly via canonical motifs in its intracellular domain. The recruitment of TRAF2 to CD40 activates key signaling pathways in B cells, including MAPK p38, PI3K/AKT/mTOR, and canonical (NF‐κB1) and non‐canonical (NF‐κB2) NF‐κB signaling (Figure 2). In contrast, TRAF3 acts as a negative regulator of NF‐κB signaling in B cells, providing a balancing mechanism. Accordingly, TRAF2 or TRAF3 deficiency results in compromised CD40 signaling in B cells, and GC formation is impaired in TRAF2‐deficient mice [63, 64]. TRAF6, which may associate directly or indirectly with CD40, contributes to distinct signaling outcomes, further diversifying the response. Through selective recruitment of specific TRAF proteins to different intracellular sites, CD40 orchestrates a finely tuned signaling network that modulates the activation of B cells [65].

Evidence indicates that the dynamics and strength of CD40 signaling are pivotal in dictating B cell fate decisions, shaping their progression into GCs or their differentiation along the EF pathway. High CD40 stimulation, often linked to strong antigen presentation by high‐affinity B cells, promotes rapid ASC formation in EF responses. Conversely, lower CD40 stimulation supports GC entry, favoring prolonged clonal expansion and Ig diversification [66, 67, 68]. In the first 2–3 days after immunization, CD40‐induced signaling is observed in antigen‐specific B cells located in the interfollicular zone and at the T‐B border (Figure 1). Extended availability of CD40 stimulation and T cell help during this early activation phase reduces the differentiation of precursor cells into the GC pathway, while transient removal of T cell help via CD40 enhances GC commitment [69]. Accordingly, cells destined for either the GC or the EF response emerge around day 3–4 [18, 55]. Indeed, during the initial 2–4 days of activation, B cells undergo multiple rounds of proliferation [69, 70] with a dynamic network of different transcription factors such as c‐Myc, IRF4 and BCL6 orchestrating the fate of precursor B cells [55, 71, 72, 73]. In mice with B cell‐specific TBK1 deficiency, BCR and CD40 activation through NF‐κB2 and PI3K/AKT is amplified, disrupting the IRF4/BCL6 balance required for GC formation. Correspondingly, while TBK1‐deficient B cells can differentiate into GC precursors following malaria infection or immunization, they fail to commit to the GC pathway, whereas EF differentiation is maintained [74].

B cell fate decisions can also be modulated by TLRs, which sense innate‐like pathogen‐associated molecular patterns (PAMPs) like nucleic acids and bacterial components [75]. TLR ligation induces receptor dimerization and recruitment of the adaptor protein MyD88, leading to the assembly of a multimeric signaling complex and activation of MAPK and NF‐κB signaling. MyD88‐dependent TLR7 and TLR9 signaling can operate in conjunction with BCR signaling, co‐opting BCR signaling pathway components like LYN and SYK for full activation. TLR stimulation can strongly support both GC responses and EF responses, the latter especially in T cell‐independent contexts. Paradoxically, whereas TLR9 activation was implicated in autoantibody formation, it also exerts a protective role against TLR7‐driven autoimmunity [75, 76].

Cytokines such as IL‐2, IL‐4, IL‐21, and IFN‐γ play critical roles in promoting B cell proliferation, CSR, and driving the differentiation of both EF B cell responses and GC B cells. Similarly, cytokines like IL‐12, IL‐2, TNF‐α, and IFN‐γ influence T helper (Th) cell polarization, indirectly shaping B cell outcomes by modulating the cytokine milieu and the type of T cell help available. For example, IL‐12 promotes Th1 polarization, supporting IFN‐γ production that enhances B cell responses to intracellular pathogens, while IFN‐γ signaling also promotes the expression of the transcription factor T‐bet and accelerates the differentiation of ASCs [35]. Indeed all types of IFN signaling, type I IFNs [20], IFN‐γ [35, 77] and IFN‐λ [78], have been shown to promote EF differentiation and are associated with the generation of autoantibodies. Thus, depending on the immunological setting, the decision between the formation of EF B cells and GC entry may be influenced by signals induced through BCR, CD40, IL‐21R, IL‐2R (CD25), TLR7, TLR9, and IFN‐γ receptors, which activate key signaling pathways, including NF‐κB, PI3K/AKT/mTOR, MAPK, and JAK–STAT signaling pathways [1, 8, 35, 75, 79, 80, 81] (Figure 2). However, the negative molecular regulators of B cell differentiation in the context of GC and EF differentiation are still not well understood.

In summary, B cells integrate a complex array of extrinsic and intrinsic cues to determine their fate. Factors such as antigen affinity, epitope density, and receptor‐ligand dynamics play significant roles. High‐affinity antigens typically drive EF differentiation, while lower‐affinity antigens guide B cells toward GC responses [53, 57, 82]. Signals like CD40L, cytokines, and TLR ligands converge with BCR signaling, collectively guiding B cells to commit to either the GC or EF pathway. This coordination ensures both immediate protection and the establishment of long‐term humoral memory. In this review, we will focus on the activation and modulation of signals that drive the fate decision between the EF and GC response. In particular, we will concentrate on PI3K/AKT/mTOR signaling modulation and the role of inhibitory signals.

## 4 Requirements for Positive and Negative Regulators in EF vs. GC Commitment During T Cell‐Dependent Humoral Responses

First, unless constitutively expressed, rapid expression of the receptors and ligands within 1–2 days after initial activation is mandatory to modulate initial B cell activation and fate decisions. For example, IL‐2 and CD25 are rapidly expressed following immunization by T and B cells, respectively [83]. Therefore, upon antigen encounter, pre‐TFH cells can instruct activated B cells via IL‐2 and the activation of the mTOR pathway to favor the EF fate. Both CD40 and IgM stimulation in mice rapidly induce the expression of the transcription factor Bhlhe40, which restrains the formation of early GC B cells [84]. Similarly, we found that FAS expression is induced within 24 h in naive human B cells following CD40 stimulation [2]. At this time point, the majority of FAS‐expressing B cells are resistant to FAS‐induced apoptosis. Instead, FAS engagement results in modulation of the mTOR axis and expression of the transcription factors BCL6 and c‐MYC, supporting GC commitment [2]. In autoimmune lymphoproliferative syndrome (ALPS) patients carrying mutations in *FAS* (ALPS‐FAS), we detected increased PI3K/AKT/mTOR activity in tissue‐resident B cells and an enhanced EF response [2], concomitant with strongly reduced GCs [2, 85]. Second, activated B cells need to be localized in close proximity with activated T cells to sense cell–cell contact‐dependent factors. During initial B and T cell activation, T‐B interactions may take hours. EF B cells in the human spleen show low CXCR5 expression and high CXCR3 expression [2]. Both activated naïve and DN2 express FAS at intermediate levels between FAS‐negative resting naïve and FAS‐high expressing GC B cells. Moreover, pre‐TFH cells (CXCR5 and PD‐1 intermediate) cells express FASL [2, 36]. Hence, we suggest that FAS–FASL interactions between recently activated B cells and pre‐TFH cells at EF sites modulate B cell fate decisions via the regulation of B cell PI3K/AKT/mTOR signaling. FAS expression in B cells, particularly in association with EF responses and low CD21 expression, has been repeatedly documented [86, 87]. However, in this context, FAS is predominantly regarded as an apoptosis‐inducing receptor. We propose that FAS may also function as an inhibitory receptor during the initial stages of B cell activation, thereby playing a regulatory role in B cell fate decisions.

Under the premise of its apoptosis‐inducing function, the role of FAS in GC selection and maintenance of humoral tolerance remains contentious [88, 89, 90]. Notably, GC B cells are very sensitive and highly susceptible to cell death in vitro even without FAS stimulation and require strong BCR and CD40 stimulation for survival [91, 92]. Despite the high expression of FAS in human and murine GC B cells, neither of their descendants, i.e., memory cells and long‐lived plasma cells, accumulate in human or murine FAS deficiency [93, 94, 95]. Instead, GC‐derived memory B cells are almost completely absent in the peripheral blood and SLOs of ALPS‐FAS patients [2]. Hence, FAS‐mediated deletion may not be required for negative B cell selection in GCs under physiological conditions. In a previous study, we analyzed patients with either a somatic *FAS* mutation or a germline *FAS* mutation and somatic loss‐of‐heterozygosity, and compared the fate of GC‐derived B cells competent or deficient for FAS signaling within the same individual [93]. FAS‐deficient memory B cells showed increased rates of SHM and specific signs of polyreactivity. Unfortunately, the CD27‐ compartment containing EF‐associated B cells could not be analyzed in this study. In healthy conditions, SHM and affinity maturation may constrain autoimmunity by mutating GC B cells away from autoreactivity [48, 49]. In our study, it was not determined whether higher mutation levels were a result of extended GC reactions or repeated GC entry of autoreactive clones. In any case, in this situation, FAS deficiency prevented removal of polyreactive cells from the memory repertoire [93]. Based on our recent discoveries [2], we propose that, independent of its apoptotic function, FAS may act as an inhibitory receptor in B cells at various differentiation stages. This role includes acting as a checkpoint regulator at the critical juncture between GC entry and commitment to the EF response through the regulation of the PI3K/AKT/mTOR axis and possibly influencing B cell fate decisions during established GC reactions. As implied above, this may explain why GC‐derived B cell populations are not accumulating in FAS deficiency. Further, as discussed below, FAS may suppress GC entry in secondary GC responses.

### 4.1 PI3K/AKT/mTOR: A Key Regulator of EF Versus GC Cell Fate Decision

The PI3K/AKT/mTOR signaling pathway plays a pivotal role in regulating B cell fate decisions, particularly in determining whether B cells follow the GC or EF pathway. High PI3K signaling favors rapid differentiation into ASCs and promotes the EF response. In fact, the rapid differentiation of low‐affinity ASCs can serve as a proxy for identifying the EF response. In lymphocytes, PI3K signaling regulates activity of AKT and the mTOR kinase, which assembles into two distinct complexes. mTOR complex 1 (mTORC1) is characterized by the Raptor subunit, and mTOR complex 2 (mTORC2) is defined by the Rictor subunit (Figure 2). PI3K‐induced PI(3,4,5)P_3_ promotes the recruitment of PH‐domain‐containing proteins such as PDK1 and AKT. Proximity of constitutively active PDK1 and AKT allows phosphorylation of AKT at Thr 308 by PDK1. Through mechanisms that are not yet fully understood, PI3K can also activate mTORC2, which phosphorylates AKT at Ser 473, further enhancing AKT activity and influencing its substrate specificity for downstream targets. Fully activated AKT is crucial for regulating mTORC1 activity, which governs cell growth, proliferation, and differentiation. In this pathway, PI3K/AKT activation leads to AKT‐dependent phosphorylation and inactivation of the mTORC1 inhibitor Tuberous Sclerosis Complex (TSC). Inhibition of TSC releases the small GTPase Rheb, which is an essential activator of mTORC1. mTORC1 in turn activates several downstream substrates including the two key effectors S6 kinases (S6Ks) and the 4E‐BP/eIF4E axis to regulate protein synthesis, cell growth, differentiation and proliferation [96, 97]. Numerous signaling pathways induced by different receptors expressed by B cells (BCR, CD40, cytokine receptors) converge on TSC, suggesting that distinct signaling pathways are integrated to control mTORC1 activity [96, 98, 99] (Figure 2). As a consequence, the regulation of mTORC1 activity may switch from PI3K dependent to PI3K independent during initial B cell activation, which renders B cells more sensitive to inhibitory or activating signaling pathways. Thus, PI3K and mTOR signaling pathways are integrated in a complex molecular framework downstream of different receptors, including growth factor receptors, to control mTORC1 activity and to shape B cell fate decisions.

### 4.2 PI3K/AKT/mTOR Pathway Dysregulation and Signal Rewiring

Dysregulation of the PI3K/AKT/mTOR pathway has been extensively studied in both mouse models and human disorders. Low PI3K activation, caused by defects in the PI3K regulatory subunit p85α or its isoforms (p85α/p55α/p50α) in mice [100, 101] or humans [102], results in impaired early B cell development in the bone marrow, leading to B lymphopenia and reduced serum Ig levels. Similarly, the deletion or inactivation of the PI3K catalytic subunit p110δ impairs BCR signaling, reduces B cell proliferation, and decreases baseline, T‐dependent, and T‐independent antibody secretion [103, 104, 105]. The regulation of PI3K/AKT/mTOR signaling plays a crucial role in determining the balance between the generation of EF and GC responses. mTORC1 inactivation in B cells, either through Raptor deletion or PKC‐β deficiency, impairs the generation of antibody‐secreting EF‐derived plasma cells [106, 107]. Conversely, hyperactivation of mTORC1 in a mouse model with B cell‐specific TSC1 deletion accelerates EF plasma cell differentiation [108]. Inactivation of mTORC2 in mouse B cells by deletion of Rictor results in reduced but not abrogated GC formation and decreased ASC formation and antibody secretion [109]. PI3K generated PI(3,4,5)P_3_ can be dephosphorylated by 3‐phosphatases to form PI(4,5)P_3_. The best‐studied 3‐phosphatase is phosphatase and tensin homolog (PTEN), which exerts an important role in B cell fate decisions [50, 60, 110, 111, 112, 113, 114]. Whereas PTEN expression is induced upon B cell activation via CD40 and BAFFR [2, 115], the regulation of PTEN activity and function in B cells is only poorly understood. In B cells, PI3K/AKT signaling negatively regulates the activity of the transcription factor FOXO1, which in turn controls expression of AID required for class switching. Mouse B cells lacking the PI3K antagonist PTEN generate increased numbers of ASCs following immunization. Additionally, hyperactive PI3Kα and PI3Kδ signaling or defective PTEN suppresses CSR through enhanced AKT activity and reduced AID expression [110, 112].

In lymphocytes, the PI3K/AKT/mTORC1 signaling network is rewired through several mechanisms. In particular, cell growth and proliferation in lymphocytes are linked to the 4E‐BP/eIF4E axis whereas S6Ks may be involved in the regulation of cell differentiation. Accordingly, the increased susceptibility of lymphocytes to the mTOR inhibitor rapamycin is associated with decreased expression of 4E‐BP1 relative to that of 4E‐BP2, which is more sensitive to rapamycin inhibition [116]. Furthermore, several studies have shown that PI3K/AKT/mTOR activity is rewired between different B cell subpopulations, with significant consequences for B cell differentiation, proliferation, and apoptosis. For example, in GC B cells, BCR and CD40 signaling are attenuated compared to naive antigen‐inexperienced B cells. Constrained BCR signaling in GC B cells was associated with increased activation of negative regulators of BCR signaling, creating a negative feedback loop [117, 118]. Similarly, CD40 stimulation is reprogrammed in GC B cells and combined CD40 and BCR signals are required to promote mTOR activation, cell cycle entry, and positive selection [92, 119]. As BCR stimulation alone leads to apoptosis or insufficient activation, CD40‐induced signals participate, dependent on the B cell differentiation stage, in the control of distinct B cell fate decisions [120, 121]. Prior to GC entry, IL‐21R signaling promotes B cell expansion and synergizes with BCR and CD40 to increase mTOR activity [122] and can potently induce autoreactive T‐bet+ cells [123]. In GC B cells, CD40 and IL‐21R together strongly induce mTOR activation to promote GC B cell positive selection and, in conjunction with BCR stimulation, support differentiation into memory B cells or long‐lived plasma cells [124].

### 4.3 Role of PI3K/AKT/mTOR Signaling in CSR

**FIGURE 3:**
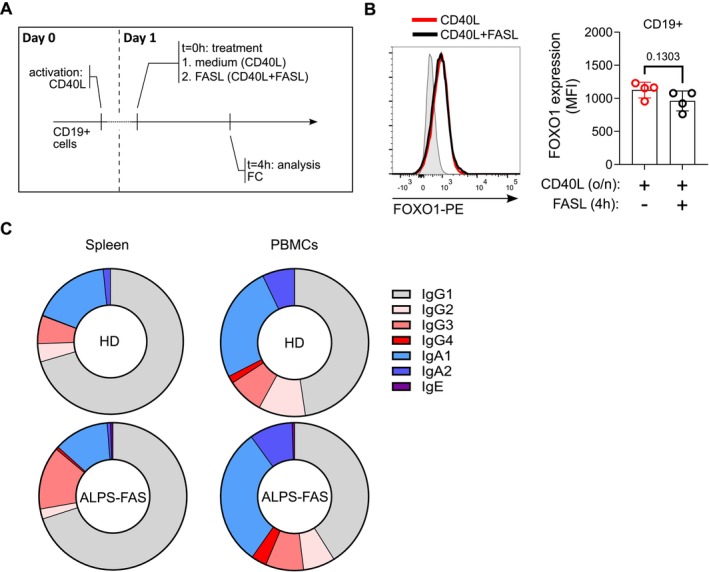
FAS signaling does not impact FOXO1 expression in healthy human B cells, and class switch recombination is not skewed to specific switched isotypes in ALPS‐FAS patients. (A) Experimental setup to study FOXO1 expression in in vitro‐activated human CD19+ B cells. Human B cells were isolated from peripheral blood of healthy donors (HDs) and activated overnight (o/n) with CD40L. CD40L‐stimulated cells were cultured for 4 h with or without 100 ng/mL FASL and analyzed by flow cytometry (FC). (B) Representative histogram of FOXO1 expression in CD19+ B cells activated overnight with CD40L ± 4‐h FASL (100 ng/mL). Gray histogram shows fluorescence minus one (FMO) control. Statistical analysis of FOXO1 MFI. Data are from four biological replicates. Mean ± SD is shown. Two‐tailed paired t‐test, p‐value is indicated. (C) Frequency of BCR isotype distribution was analyzed by flow cytometry in spleen and PBMCs of HDs and ALPS‐FAS patients. The distribution of indicated switched immunoglobulin subclasses in relation to IgD‐, IgM‐ CD19+ B cells in indicated tissues of HDs and ALPS‐FAS patients is displayed. The mean was calculated from PBMC B cells of 11 HDs, 11 ALPS‐FAS patients (age‐matched), and splenic B cells of 7 HDs and 4 ALPS‐FAS patients.

EF responses have the capacity to produce both unswitched B cells and class‐switched B cells. Since CSR can take place outside of GCs, initiation of CSR and the decision for a B cell to either enter the GC or differentiate at EF areas may occur at very similar time points [69, 125] (Figure 1). In fact, expression of AID transcripts and appearance of GLTs were detected around 24 h before cells with unequivocal commitment to the GC or the EF response appeared [18]. PI3K/mTORC2/AKT/FOXO1 signaling promotes ASC formation and antagonizes CSR, whereas mTORC1 activity is required for both ASC and CSR [126]. Since mTORC2 operates upstream of AKT and is therefore involved in the regulation of the AKT‐FOXO1 signaling axis, which is critical for CSR, these findings suggests that precise regulation of PI3K/AKT, mTORC1, and mTORC2 activity is required at different levels to regulate B cell differentiation. Subtle differences in the requirements for PI3K/AKT and mTOR activity between CSR, EF differentiation, and GC entry suggest the existence of regulatory networks that uncouple these fate decisions during B cell activation. Given that receptors such as BCR, CD40, and TLRs transmit overlapping signals driving GC, EF, and CSR fates, it is critical to understand how downstream signaling pathways are regulated to accurately direct and coordinate these distinct outcomes. In our studies we observed enhanced mTOR and BCR signaling in tissue‐resident B cells of FAS‐deficient ALPS‐FAS patients. Using in vitro models, we discovered that in healthy naive B cells transient FAS stimulation dampens CD40‐induced PI3K/AKT/mTOR activity [2]. We observed reduced phosphorylation of mediators that are associated with mTORC1 (AKT T308, pPDK1, pS6) and mTORC2 (AKT S473) activity after 4 h of FAS stimulation. In B cells, active AKT may phosphorylate FOXO1, resulting in its nuclear exclusion and degradation and conversely, FOXO1 accumulates in absence of AKT signaling. Despite reduced AKT phosphorylation, we did not observe significant changes in FOXO1 expression in CD40‐activated B cells after FAS stimulation (Figure 3A,B). *De novo* FOXO1 protein synthesis may require more time than the 4–6 h after which we analyzed the cells in our system. Further, in our experiments, FAS‐induced downmodulation of mTOR activity was more dominant for mTORC1‐associated signaling intermediates, presumably via the nuclear exclusion of endogenous PTEN. Thus, it is possible that the reduction in AKT S473 phosphorylation induced by FAS stimulation, and consequently the diminished activity of the mTORC2 pathway, was insufficient in CD40‐prestimulated cells. As a result, FOXO1 may have been stayed sequestered in the cytoplasm and subjected to degradation. In line with these data, we observed a normal distribution of class‐switched isotypes in the few memory B cells generated in ALPS‐FAS patients (Figure 3C). Notably, PTEN‐mediated activation of FOXO1 promotes IgD expression, which is required for efficient GC formation [50]. Thus, further experiments should firmly establish the role of FAS in controlling FOXO1 activity and IgD expression. Future studies could address whether FAS is involved in shaping the substrate specificity of AKT in recently activated B cells, thereby contributing to B cell fate decisions. Notably, differential AKT phosphorylation was shown to affect AKT substrate specificity in GC B cells [118].

### 4.4 Altered mTOR Signaling Influencesand EF B Cell Development: mTOR Inhibitors and IEIs

**FIGURE 4:**
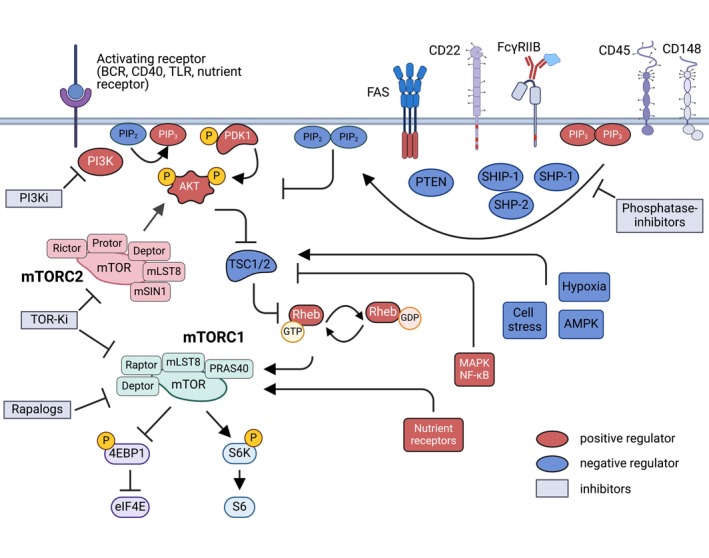
Inhibitory receptors modulate the PI3K/AKT/mTOR pathway to influence B cell fate decisions. Upon activation, B cells express several inhibitory or regulatory receptors, such as CD22, FcγRIIb, CD45, CD148, and FAS. These receptors are often associated with, or induce the activity of phosphatases like SHIP‐1, SHP‐1, SHP‐2, or PTEN, which counteract PI3K activity. A critical nodal point in this regulation is the mTORC1 inhibitor TSC complex (TSC1/TSC2), where multiple signaling pathways converge. Alternative activation can induce mTORC1 and mTORC2 activity, which are also subject to distinct regulatory mechanisms. Specific inhibitors can target different components of these signaling pathways, and those discussed in the text are indicated. Positive regulators are shown in red, negative regulators in blue, and pharmacological inhibitors in magenta. Phosphorylation of specific intermediates is highlighted in yellow. Arrows in the figure represent cross‐talk between different pathways, with a particular focus on the regulation of the PI3K/AKT/mTOR pathway.

Using mTOR inhibitors, several reports provide compelling evidence that B cell‐intrinsic regulation of the mTOR pathway is required for B cell proliferation, selection, and differentiation [106, 119, 127, 128, 129]. The allosteric mTOR inhibitor rapamycin forms a complex with FKBP12, resulting in mTORC1 inhibition and, upon prolonged exposure, inhibition of mTORC2 activity. ATP‐competitive mTOR kinase inhibitors (TOR‐KIs) were developed to block the activity of both mTORC1 and mTORC2 to overcome the limitations of rapamycin in the usage as anticancer treatments (Figure 4). While high concentrations of both rapamycin and TOR‐KIs suppress B cell proliferation and ASC differentiation, only rapamycin constrains CSR whereas TOR‐KIs promote it [126]. Importantly, the suppressive function of rapamycin on CSR occurs independently of its effects on proliferation, as even rapamycin concentrations permissive to B cell proliferation still suppress CSR. As described above, the PI3K/AKT pathway was previously described to suppress CSR [110, 112]. In addition, mTORC1 appears to post‐transcriptionally regulate the expression of AID [130], illustrating the need for a better understanding of how these pathways are involved in the regulation of B cell differentiation. In addition to the magnitude of mTORC1 and mTORC2 activity required for distinct B cell differentiation pathways, the timing of mTOR regulation is also essential. For example, in B cells already committed to cell division, inhibition of mTORC1 activity allows proliferation [119] while CSR is prevented [130]. These studies conclusively demonstrate the need for dynamic and temporal regulation of both mTORC1 and mTORC2 activity in the regulation of B cell proliferation, antibody secretion, and CSR. However, they mostly relied on genetic and pharmaceutical tools, which inherently generate extremes of signaling pathway activation or inhibition (genetic deletion or overexpression of signaling pathway components) or do not mirror physiological regulatory mechanisms (chemical inhibitors) of signaling networks, respectively. Thus, additional studies will be required to shed light on how B cells integrate signals from different receptors to regulate PI3K/AKT, mTORC1, and mTORC2 signaling in the control of B cell fate decisions.

Transient treatment of mice with TOR‐KIs prior to and during immunization protocols improved humoral T cell‐dependent immune responses, resulting in increased Ig titers, higher affinity of antigen‐specific class‐switched antibodies, and a higher frequency of memory B cells [126]. In mice vaccinated with live‐attenuated strains of *Salmonella Typhimurium* , the formation of GCs is delayed and the humoral immune response is dominated by EF responses accompanied by the generation of IgM and IgG2c antibodies. When mice were treated with TOR‐KIs 3–6 days after *S. Typhimurium* vaccination, a significantly reduced early production of IgG2c antibodies was observed. Additionally, a significant increase in antigen‐specific IgM antibodies and higher levels of IgG3 antibodies were found, suggesting that TOR‐KIs promoted the generation of GCs while reducing EF differentiation. Similarly, transient TOR‐KI treatment could promote the generation of antigen‐specific antibodies in aged mice that are naturally showing signs of decreased humoral responses [126]. In ALPS, rapamycin treatment rapidly resolves autoantibody‐dependent cytopenias without inducing hypogammaglobulinemia [131, 132]. It is possible that restored GC selection contributes to this phenotype. Nevertheless, further studies are needed, and it would be highly intriguing to investigate the development of self‐reactive B cells alongside the emergence of self‐reactive antibodies in the presence or absence of transient mTOR inhibitors. This approach could provide valuable insights into how mTOR signaling influences B cell fate decisions and the progression of autoimmunity.

In humans, activated PI3K‐δ syndrome (APDS), caused by mutations in PIK3CD and PIK3R1, is characterized by hyperactive PI3K signaling and associated with defective CSR, impaired memory B cell formation, and enhanced ASC differentiation [133, 134]. Similarly, PTEN deficiency in humans leads to CSR defects, emphasizing the importance of balanced PI3K activity in B cell responses [135]. Aberrant PI3K/AKT signaling has also been implicated in autoimmunity. Mice overexpressing CD19 [136, 137], which amplifies PI3K activation, develop autoantibody‐mediated autoimmune disorders due to a breakdown in tolerance mechanisms. In ALPS‐FAS, signaling via the PI3K/AKT/mTOR pathway is dysregulated, particularly in EF‐related B cell subsets [2]. This dysregulation is associated with a hyperactive B cell activation state and enriched EF B cell differentiation. Interestingly, naive peripheral blood B cells (IgD+ CD27−) from ALPS patients show no significant mTOR hyperactivation. These findings emphasize the importance of tissue‐specific analyses, as variations in architecture and microenvironment likely influence signaling dynamics.

### 4.5 Inhibitory and Modulatory Receptors Regulate PI3K/AKT/mTOR Signaling to Govern B Cell Fate Decisions

PI3K/AKT/mTOR signaling needs to be tightly regulated during initial B cell activation to balance GC and EF responses. Multiple receptors, including BCR, CD40, and TLRs, drive PI3K activity with varying intensities, while inhibitory receptors fine‐tune this activation to modulate B cell responses (Figure 4). Despite this, the specific receptors governing PI3K activity and the mechanisms regulating PI3K and PTEN during early B cell activation remain poorly understood. B cells express several inhibitory receptors [138]. Here, we will discuss the role of inhibitory receptors, including Fcγ receptor IIB (FcγRIIB), members of the sialic acid‐binding immunoglobulin‐like lectin (Siglec) family, and FAS in the context of humoral tolerance.

The low‐affinity IgG inhibitory FcγRIIB plays a critical role in negatively regulating BCR‐mediated signals in B cells, contributing to immune tolerance. Co‐ligation of FcγRIIB with the BCR results in the LYN‐dependent phosphorylation of the cytoplasmic immune receptor tyrosine‐based inhibitory motif (ITIM) encoded by FcγRIIB. Subsequently, phosphatases like Src homology 2‐containing protein tyrosine phosphatase‐1 (SHP‐1, encoded by *PTPN6*) and SH2‐domain‐containing inositol phosphatases (SHIPs), in particular SHIP1 (encoded by INPP5D), are recruited to the plasma membrane. Here, SHP‐1 and SHIP‐1 convert PI(3,4,5)P_3_ to PI(3,4)P_2_, preventing the recruitment of activating PH‐domain‐containing proteins like AKT and BTK to the plasma membrane, thereby inhibiting BCR‐induced signaling. Additional inhibitory receptors are expressed by B cells, including sialic acid‐binding immunoglobulin‐like lectin (Siglec) family members CD22 and Siglec‐G, and CD72. Similar to FcγRIIB, these receptors carry cytoplasmic ITIMs that, following phosphorylation, allow the recruitment of phosphatases and thereby inhibition of BCR signaling. Accordingly, natural genetic variants of *CD22* in humans or CD22 deletion in mice predispose to autoimmunity [139, 140, 141]. Reduced or absent FcγRIIB expression is linked to autoimmunity in both humans and mice. FcγRIIB‐deficient mice often develop high serum IgG antinuclear antibodies (ANAs) and strain‐specific nephritis as they age. Studies have highlighted the tolerogenic role of FcγRIIB in regulating GC B cell and plasma cell differentiation [142, 143, 144, 145]. For example, FcγRIIB helps to exclude autoreactive B cells from GC reactions, reducing autoantibody production by GC‐derived plasma cells [146]. It also regulates the magnitude of short‐lived plasma cell responses [147], preventing an uncontrolled expansion of autoreactive IgG+ plasma cells in the spleen, and was implicated in the control of long‐lived plasma cell persistence in the bone marrow [145]. In the absence of FcγRIIB, autoreactive and polyreactive IgG+ B cells actively participate in GC responses and undergo SHM, producing highly autoreactive antibodies [144]. Notably, the spontaneous nature of autoimmunity onset in FcγRIIB‐deficient mice, which worsens with age, restrained many studies in their interpretation of whether autoantibodies were derived from plasma cells of GC or EF origin. In mice carrying natural occurring FcγRIIB promoter variant knock‐ins, GC B cells exhibit impaired FcγRIIB upregulation [148, 149]. This impairment was associated with enhanced GC formation, increased affinity maturation, and autoantibody production. Notably, immunization in these models with exogenous antigen resulted in the activation of autoreactive clones and rapid but transient autoantibody generation [148], suggestive of an increased EF response. These observations implicate that FcγRIIB regulates EF versus GC fate decisions. Additionally, the generation of autoantibodies was amplified in recall responses, suggesting that autoreactive memory B cells, generated in a primary response, may participate in secondary immune responses [148, 149]. Whether these autoreactive cells were initially formed in the GC or via EF differentiation and whether the amplified secondary response was due to their recruitment into GCs or due to their expansion at EF sites was not further studied. It will be important to understand if EF‐derived effector cells can enter GCs at later time points or during recall responses and how their access to GC is regulated.

B cells involved with EF responses in chronic infection, immunodeficiency, and autoimmunity exhibit considerable heterogeneity, with their differentiation and functional characteristics being influenced by the distinct immunological settings in which they arise [150, 151]. Correspondingly, atypical CD21‐ memory cells in malaria, DN2 B cells in SLE, and exhausted memory B cells in HIV infection exhibit distinct expression patterns inhibitory receptors that may antagonize secondary activation [151]. Thus, in contrast to atypical CD21‐ cells observed in malaria infection, BCR stimulation is conserved in SLE‐associated DN2 B cells despite enhanced expression of inhibitory receptors such as FCRL5, CD22, and FcγRIIB, along with lower BCR expression. In autoimmunity and immunodeficiency, high SYK expression results in constitutive activation of CD21low B cells, but additional BCR stimulation is compromised [152]. Thus, since BCR stimulation can be differentially affected in EF B cells generated in various immunological settings linked to expanded EF responses, reactivation and differentiation of distinct EF‐associated B cell populations may vary between conditions.

In our experiments, we observed that FAS predominantly regulated mTOR activity in naïve B cells after initial CD40 activation, whereas CD27+ memory B cells, mostly expressing switched BCR isotypes, were less affected [2]. Our data suggest that GC‐derived CD27+ memory B cells may not require T‐dependent regulatory signals to guide bifurcating fate decisions. Instead, soluble FASL stimulation may promote ASC differentiation in memory B cells when T‐cell help is limiting [153]. It is conceivable that FAS stimulation also regulates BCR‐induced signaling. In fact, in vivo B cells will collect antigen via their BCR prior to receiving T cell help and CD40 triggering. However, BCR stimulation alone is a very weak inducer of FAS expression, whereas CD40 stimulation is critical for the robust upregulation of FAS, thereby enhancing B cell susceptibility to FAS engagement. Thus, only B cells that acquired, processed, and presented antigen via MHCII to cognate antigen‐specific T cells will be subjected to the putative FAS checkpoint of B cell activation. Consistent with observations of reduced CD40‐induced mTOR activity following FAS stimulation [2], FAS may regulate responses to subsequent antigen encounters by modulating BCR‐induced signaling. In GC B cells, BCR and CD40 signaling are rewired compared to naive B cells due to higher phosphatase expression in GC B cells. This leads to reduced PI3K/AKT/mTOR activity downstream of CD40 and diminished BCR‐induced activation of SYK/BTK, mTORC1, and MAPK signaling pathways. However, CD40‐induced NF‐κB signaling remains intact in GC B cells. Thus, B cells integrate signals from various receptors to fine‐tune humoral responses. It is likely that similar signal integration occurs during initial B cell activation, and regulatory mechanisms, such as FAS stimulation, contribute to signaling modulation. Furthermore, the analysis of different signaling pathways is important because different arms of CD40 or BCR activation can be differentially affected by regulatory networks.

## 5 Memory B Cells and the EF Response

The contribution of GC‐derived memory B cells to secondary GCs is under debate [154, 155, 156]. Apart from extrinsic factors, such as negative feedback by pre‐existing antigen‐specific serum antibodies, participation of GC‐derived memory B cells in secondary immune responses may depend on intrinsic factors like antigen affinity, BCR isotype usage, SHM status, germline affinity, and expression of inhibitory receptors [157]. In murine secondary responses induced by homologous re‐immunization, GCs are dominated by naïve B cells and antigen‐specific memory B cells with low somatic mutations. In such a situation, re‐diversification of highly mutated memory B cells in GCs is rare [158, 159, 160]. In humans, pre‐existing cross‐reactive memory B cells can be recruited to secondary GCs where they may undergo further affinity maturation [161, 162]. Nevertheless, cross‐reactive memory B cells appear to dominate the rapid ASC response, and naïve antigen‐inexperienced B cells, targeting novel epitopes, are preferably recruited into recall GCs and improved through clonal selection and affinity maturation [162, 163, 164]. Notably, although the antibody binding strength of memory cells was not higher compared to naïve cells in the study by Li et al., memory B cells preferably differentiated into ASCs, suggesting that antigen affinity was not determining B cell fate [164]. Instead, GC‐derived memory B cells intrinsically have a higher propensity to differentiate into ASCs, which could be due to their decreased activation threshold [165, 166]. In fact, different mechanism have been proposed that result in enhanced responses of memory B cells as compared to naïve B cells [167, 168, 169, 170]. Lineage tracing experiments suggest that memory B cells can be generated outside of GCs continuously through an immune response [171]. These memory cells carry few somatic mutations and rarely express switched isotype BCRs but can exhibit measurable antigen affinity. Based on the similarity of their phenotype (IgM+ CD80+) to memory B cells that were previously described to participate in secondary GC responses, these data suggest that GC‐independent memory B cells may indeed reenter secondary GCs. Whether the preferred reengagement into new GCs could be dependent on antigen affinity and as a consequence on BCR signaling strength or on T cell help, i.e., CD40 signaling strength, is unknown. How B cells that were generated in a primary immune response via the EF pathway without or only very little SHM and no access to clonal selection are behaving in secondary responses in these settings is unknown.

In SLE‐associated DN2 B cells, but not their immediate precursors (activated naïve B cells), CD40 stimulation is impaired [12]. Moreover, in SLE patients activated naïve, DN2 B cells and their end descendant ASCs show low levels of SHM compared to GC‐derived memory B cells, suggesting that they have not undergone stringent or iterative affinity maturation in GCs [12, 172]. At the same time, DN2 exhibits higher expression of HLA‐DR and CD86, which are important for antigen‐presentation and T–B interaction [12], and CD21low B cells can become competent antigen‐presenting cells upon activation [87]. Atypical B cells were dispensable for humoral responses following immunization with Plasmodium sporozoite but were required for GC maintenance, likely by their superior antigen uptake and presenting function [173]. In HIV‐infected patients, antigen‐specific T bet+ B cells are expanded and are clonally related to GC B cells but have lower SHM levels and poor affinity maturation [174]. It is thus conceivable that in certain situations, B cells that differentiated in a primary immune response at EF sites may modify their receptor expression profile in secondary responses (e.g., expression of CXCR5, EBI2, and CXCR3), enabling them to relocate to B cell follicles. This repositioning may facilitate access to T cell help in recall responses and allow entry into GCs to diversify their BCR variable regions. Thus, whether EF‐generated B cells can be diversified via the GC remains to be determined, but the functional changes (autoreactivity, longevity) that can be imposed during such processes warrant further investigations.

## 6 Inhibitory Signals: Guardians for Bystander Activation?

Antigen encounter represents the initiation of B cell activation in primary and secondary humoral immune responses. For example, reactivation of memory B cells is primarily mediated by specific antigens and interactions with specific TFH cells [175]. Nevertheless, B cells can be activated in a bystander antigen‐independent manner, primarily driven by the inflammatory environment and the presence of cytokines during an immune response. In humans, bystander reactivation of memory B cells has been implicated in memory renewal and maintenance [176], and in pediatric cohorts vaccination or infections result in the increase of antibody titers for unrelated specificities [177]. On the contrary, in mouse models, viral infection is sufficient to activate specific memory in the absence of specific or bystander T cell help [178], and bystander proliferation of non‐related specificities is a rare event even during vaccination or TLR stimulation conditions [179].

Bystander activation of antigen‐inexperienced naive B cells can contribute to amplifying and accelerating the immune response in the initial activation phase. This may enable naïve B cells with very low affinity or undetectable antigen‐binding to participate in immune responses, leveraging the diversity of the host BCR repertoire. Bystander activation can be associated with enhanced availability of activating cues or by enhanced B cell‐intrinsic activation. Enhanced TLR7 activity predisposes to SLE and increased EF responses, while TLR8 and TLR9 signaling are protective (reviewed in [76]). In fact, in human B cells, BCR stimulation is not required while CD40L and IL‐21, mimicking T‐cell help, are sufficient to induce CSR and ASC differentiation [180]. While beneficial in certain situations, bystander polyclonal activation can also contribute to the production of autoantibodies [181, 182, 183]. In COVID‐19, the emergence of autoantibodies targeting a broad spectrum of antigens has been reported [33, 184, 185]. These autoantibodies exhibit variable temporal dynamics, including stable patterns (persisting for more than 12 months), transient patterns (declining within 6 months), and delayed appearance of persisting autoantibodies, the first and the latter suggesting a GC origin [185]. Molecular mimicry can contribute to the generation of antibodies in the frame of viral infection [186, 187]. However, since a corresponding pathogen molecule has not been identified for every autoantibody specificity, molecular mimicry alone does not fully account for the range of self‐reactivity observed. Bystander activation in absence of selection pressure in the EF response in an inflammatory environment can explain the generation of self‐reactive antibodies. The short half‐life of a portion of the autoantibodies support their EF origin [184]. This is in line with single‐cell repertoire studies in patients with mild and severe COVID‐19 disease, showing expansion of a naive‐derived, low‐mutation IgG1 population of antibody‐secreting cells (ASCs), reflecting features of low selective pressure [33]. These features are linked to progressive autoreactivity, targeting nuclear antigens and carbamylated proteins 10–15 days after symptom onset. The low‐selection compartment shows clonotypes recognizing both SARS‐CoV‐2 and autoantigens, including pathogenic glomerular basement membrane autoantibodies. Upon disease recovery, this pathway contracts, tolerance is restored, and acute‐derived ASCs are lost [33]. Hence, we envision that in a highly inflammatory, cytokine‐driven environment, B cells can attain sufficient PI3K/AKT/mTOR signaling to rapidly differentiate into ASCs at EF sites, largely independent of antigen affinity and without being subjected to GC‐associated selection pressures. Following this hypothesis, in situations where T cell help is not limited upon initial activation, the EF response should be expanded, leading to the production of autoantibodies. This is the case in IEIs such as CTLA‐4 deficiency or LRBA deficiency, where T cells are hyperactivated. Here, an expansion of the EF response and the generation of spontaneous GCs is observed [188, 189, 190, 191]. This phenotype is reverted by the use of CTLA‐4 Ig (Abatacept) that restores normal T cell activation [188, 192]. In the case of B cell lymphopenia, T cell activation and, in general, stimulants and cytokine activation are not limiting. In fact, in patients with IEIs that impair early B cell development, such as hypomorphic *RAG* mutations, a relative expansion of the EF response accompanied by the generation of autoantibodies is observed [193]. We speculate that in the context of a reduced B cell pool, competition for activating signals among B cells may be diminished, favoring a rapid EF response. Furthermore, in cases of B cell lymphopenia accompanied by low serum immunoglobulin levels, the negative feedback mechanisms mediated by serum antibodies are likely attenuated or absent. This reduction in feedback inhibition may further decrease competition between newly activated B cells, thereby facilitating activation and EF differentiation of B cells expressing autoreactive BCRs or those with low or negligible affinity.

In IEIs where the genetic mutation results in enhanced intrinsic B cell activation, an imbalance between the EF and GC responses is often observed. This is typically associated with a break of humoral tolerance and the generation of autoantibodies. This is the case in APDS patients displaying hyperactive PI3K/mTOR signaling pathways [133, 134] and in ALPS‐FAS patients where defective FAS signaling results in increased PI3K/AKT/mTOR [2]. Furthermore, in addition to impairing CSR, PTEN deficiency is linked with autoantibody formation in humans [194]. In mouse models, failure to downregulate PI3K/AKT/mTOR signaling in the presence of T cells helps is sufficient to drive the development of autoantibodies [195]. In mice carrying natural occurring FcγRIIB promoter variant knock‐ins, GC formation was increased [148, 149]. However, the number of antigen‐specific GC B cells remained comparable to wild‐type controls. Immunization activated autoreactive clones, leading to rapid autoantibody production and GC B cells targeting irrelevant antigens. How FcγRIIB controls BCR signaling and/or antigen uptake, processing, and presentation via MHC‐II complexes to receive help from T cells remains to be determined. Nevertheless, these observations suggest that in the absence of inhibitory receptors, bystander B cell responses become dysregulated, leading to the unintended activation and recruitment of B cells into GC or EF responses. B cells prone to bystander activation either possess an intrinsically reduced activation threshold or are polyreactive, which increases their chances of encountering antigens. This leads to the activation of B cells with a broad range of antigen reactivity. In this context, we propose that FAS acts as a novel regulatory player [2], contributing to the control of premature activation and preventing unspecific humoral responses. Another mechanism by which bystander activation of B cells may occur is through trogocytosis, the acquisition of BCRs via direct membrane transfer from adjacent antigen‐specific B cells. This process allows bystander B cells to rapidly acquire antigen receptors, and enhance antigen presentation engagement [196]. This process can promote the break of humoral tolerance to self‐antigens, as autoreactive B cells, by acquiring a BCR specific for a viral antigen, can boost the response to self‐antigen by presenting the viral antigen to the T cells [197].

## 7 FAS Signaling Guides B Cell Fate Decisions

FAS‐induced signaling is regulated at multiple levels, including ligand conformation, receptor expression and its localization in plasma membrane nanodomains, intracellular signaling hubs and mitochondrial networks. Upon binding to its membrane‐bound ligand FASL, pre‐assembled FAS oligomerizes and recruits the death‐inducing signaling complex (DISC) via DD‐ and DED‐containing adaptor proteins and caspases. The DISC composition varies depending on the cell type, activation, and differentiation status and may include FAS, the adaptor protein FADD, caspase 8, caspase 10, and c‐FLIP, a key regulator of FAS signaling. DISC recruitment triggers homodimerization and auto‐proteolytic cleavage of pro‐caspase 8 into active caspase 8 that is released into the cytoplasm. FAS‐induced apoptosis proceeds via two pathways downstream of caspase 8. In type I cells, caspase 8 activation directly cleaves and activates effector caspases, leading to cell death. In type II cells, reduced DISC formation necessitates integration with the mitochondrial BCL‐2 family network. In these situations, receptor engagement results in lower caspase 8 activation, which can cleave the pro‐apoptotic BCL‐2 family member BID into tBID, which induces mitochondrial cytochrome c release and activation of caspase 9, creating a feedback loop by cleaving caspase 8 and caspase 3. These pathways are interconnected, with significant cross‐talk [198, 199]. Ligand type is critical, as membrane‐bound FASL is typically associated with apoptosis induction, whereas soluble FASL lacks cytotoxicity, can antagonize membrane‐bound FASL function, and promote cellular differentiation and migration [199, 200]. At the membrane, FAS localization to lipid rafts is essential for efficient apoptosis induction, a process facilitated by posttranslational modifications such as palmitoylation and dephosphorylation [201, 202]. Further, the regulation of the DISC involves c‐FLIP, which can, depending on its isoform and its stoichiometric relationship with caspase 8, either inhibit apoptosis or promote NF‐κB signaling [203]. Protection against FAS‐mediated apoptosis relies on CD40‐induced PI3K activation and BCR crosslinking or simultaneous stimulation of both receptors. Resistance of FAS‐induced apoptosis also depends on NF‐κB transcription factors and the presence of c‐FLIP, predominantly expressed in three isoforms: c‐FLIP long (c‐FLIP_L_), c‐FLIP short (c‐FLIP_S_), and c‐FLIP Raji (c‐FLIP_R_) [199, 204]. In addition, BCR stimulation induces expression of FAS apoptotic inhibitory molecule (FAIM), which may protect antigen‐specific and/or high‐affinity B cells from FAS‐induced apoptosis during cognate interactions with FASL expressing T cells [205]. Finally, shortly after FAS stimulation, a second signaling complex (complex II) is assembled, which includes DED‐containing proteins but not FAS. Complex II, common to death receptors like TRAIL‐R1, TRAIL‐R2, and TNFR1, is thought to regulate caspase activation and uncouple apoptotic from non‐apoptotic signals [204].

In fact, besides apoptosis, FAS can mediate non‐apoptotic functions, including activation, proliferation, migration, and differentiation [199, 200, 206]. We propose that FAS functions as molecular switch in initial B cell activation by regulating the intensity of PI3K/Akt/mTOR activity, thereby guiding the B cells into the GC [2]. In ALPS, enhanced PI3K activation correlates with expansion of the EF response and the presence of autoantibodies [2, 207]. Understanding the molecular mechanism that mediates the non‐apoptotic function of FAS can serve to identify further switches that can be targeted to manipulate B cell maturation. In ALPS patients, FAS mutations impair non‐apoptotic FAS signaling. This defect may dysregulate mTOR activity in naïve B cells, promoting the EF responses and excessive expansion of autoreactive B cells. We observed an expansion of the EF response in ALPS with FAS mutation. The mechanistic model underlying these observations suggests that FAS acts as a regulator of PI3K signaling via PTEN. In this model, PI3K modulation is mediated by the regulation of PTEN function, operated by FAS via DAXX and USP7, a deubiquitinase that targets PTEN. Interactions between USP7 and DAXX are well established across various cell types, including murine B cells [208, 209, 210, 211, 212]. Despite its many interaction partners, the precise function of DAXX remains unclear. Initially identified as a FAS‐interacting protein that enhances FAS‐induced apoptosis [213], studies on DAXX have yielded conflicting results, with some showing it promotes FAS‐induced cell death [214], while others report the opposite [215, 216, 217]. Interestingly, a DAXX transgenic mouse model showed suppressed CD40‐induced proliferation and FAS‐induced apoptosis [216]. In EBV‐immortalized B cell lines, we observed DAXX recruitment to FAS upon stimulation, which was impaired by ALPS‐associated FAS mutations [2]. DAXX may bind to the FAS DD independently of FADD [218], potentially facilitated by caspase 8 activity during DISC formation. While our studies showed PTEN translocation to the cytoplasm upon FAS engagement, further experiments are needed to confirm whether USP7 regulates PTEN ubiquitination and localization following FAS stimulation in a DAXX‐dependent manner.

## 8 Concluding Remarks

**FIGURE 5:**
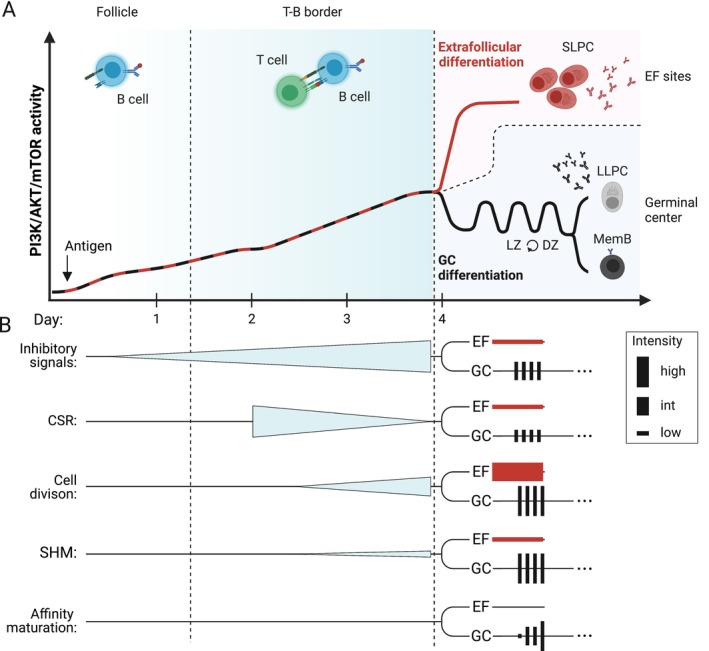
The intensity of PI3K/AKT/mTOR signaling guides B cell fate decisions during initial activation. This scheme summarizes our model. (A, B) Upon encountering an antigen, BCR engagement induces an initial activation of PI3K signaling. High‐affinity BCRs drive rapid differentiation into the extrafollicular (EF) response. Between days 2 and 3 of activation, with the help of T cells and additional signals, PI3K/AKT/mTOR signaling is further amplified. At this stage, high PI3K/AKT/mTOR signaling promotes EF differentiation, while inhibitory signaling, by modulating PI3K intensity, favors germinal center (GC) fate. Class switch recombination (CSR) is initiated and predominantly occurs in the EF space, while somatic hypermutation (SHM) can occur in the EF space but is more prominent in the GC. Proliferation begins outside the GC but becomes massive within the GC. Affinity maturation is a hallmark of the GC. In (B), processes associated with GC fate are shown in black, while those linked to EF fate are shown in red. The thickness of the bars represents the relative importance of each process in the respective populations. For the GC fate, intermittent bars indicate the occurrence respective features in reiterative transitions between the GC light zone and dark zone. SLPC, short‐lived plasma cell; LLPC, long‐lived plasma cell; MemB, memory B cell.

In summary, our studies on ALPS, combined with data from the literature, suggest a model where the intensity of PI3K signaling upon initial B cell activation determines the B cell fate decision between the EF and GC responses (Figure 5). The intensity of PI3K signaling is influenced by positive signals, such as BCR engagement, CD40, TLRs, or cytokine signaling, which are modulated by inhibitory or regulatory signals, including FcγRIIB, CD22, and FAS. The order, distribution, duration, and affinity of these signals collectively contribute to the net signaling outcome. Upon initial B cell activation, high PI3K signaling promotes EF cell fate, leading to the rapid differentiation of antibody‐secreting cells, which predominantly produce IgM isotype antibodies. However, CSR can also occur outside the follicle, allowing for the production of other antibody isotypes within the EF response. Conversely, when B cells exhibit increased sensitivity to inhibitory receptors—such as when BCR stimulation is insufficient—PI3K signaling is reduced. This reduction facilitates the migration of B cells into the GC, where SHM and affinity maturation enhance the antibody response. An imbalance in the PI3K/AKT/mTOR pathway favors EF B cell development, impairs selection processes, and predisposes individuals to autoimmunity. While our model requires further investigation, it holds potential for manipulating the intensity of PI3K/AKT/mTOR signaling to guide the type of memory generated during vaccination or in therapeutic interventions for autoimmune diseases.

## Conflicts of Interest

The authors declare no conflicts of interest.

## Data Availability

The data that support the findings of this study are available from the corresponding author upon reasonable request.
